# Papillary carcinoma of the thyroid: methylation is not involved in the regulation of MET expression

**DOI:** 10.1038/sj.bjc.6601988

**Published:** 2004-07-20

**Authors:** S Scarpino, A Di Napoli, M Rapazzotti-Onelli, E Pilozzi, L Ruco

**Affiliations:** 1 Dipartimento di Diagnostica di Laboratorio e Patologia, II Facoltà di Medicina e Chirurgia, Ospedale Sant'Andrea, Via di Grottarossa 1035-1039, 00189 Università ‘La Sapienza’, Rome, Italy

**Keywords:** DNA methylation, Met expression, papillary carcinoma of the thyroid

## Abstract

Hypomethylation has been reported to be responsible for the activation of several oncogenes. The possibility that hypomethylation is involved in the regulation of MET transcription was investigated through the analysis of the methylation status of one CpG island containing 43 CpGs in six cases of papillary carcinoma, in the corresponding normal thyroid tissue, and in two cases of hyperplastic goitre. Evidence of methylation was not found in any of the analysed CpG.

The Met oncogene encodes a trans-membrane tyrosine kinase identified as the receptor for hepatocyte growth factor (HGF) (Giordano *et al*, 1989; Naldini *et al*, 1991). Immunohistochemical studies have demonstrated that Met protein is intensely expressed in tumour cells of >95% cases of thyroid papillary carcinoma. In tumour tissue, the levels of RNA transcripts for MET are 10–100-fold higher than in the surrounding normal thyroid (Di Renzo *et al*, 1992). Gene expression studies have demonstrated that MET gene is one of the 23 genes, which are significantly more expressed in papillary carcinoma (Huang *et al*, 2001). Mutations, amplifications or other alterations of the MET gene have not been recognised, raising the possibility that an aberrant transcriptional regulation may play a critical role in gene activation. Evidence for activation of oncogenes by specific gene demethylation in cancer has been reported in the body of several oncogenes including cMYC, c-JUN, HOX11 and H-RAS (Vachtenheim *et al*, 1994; Watt *et al*, 2000).

Since the MET promoter is a 697 bp 5′-untranslated region that contains a typical CpG island spanning, with a frequency of CpGs 10 times greater than in the total gene (Accession no. Z26936), in the present study, we have explored the possibility that an altered methylation status of the MET promoter is involved in the abnormal expression of Met protein in papillary carcinoma.

## MATERIALS AND METHODS

### Immunohistochemistry

Expression of *Met* protein was investigated in 137 cryopreserved samples of thyroid tissue involved by various pathological conditions. Fragments of fresh tissue were embedded in optimal cryopreserving tissue (OCT) compound (Miles, Elkhart, IN, USA), snap-frozen in liquid nitrogen, and stored at −80°C until sectioning. Met protein was demonstrated with the DO-24 mouse monoclonal antibody.

### DNA extraction

For DNA extraction, 40 cryostat sections 10 *μ*m each were cut from six cases of papillary carcinoma of the thyroid (two follicular and four usual-type papillary carcinoma; female : male ratio was 5 : 1, and the mean age was 45 years), from the corresponding peritumoral normal thyroid tissue, and from two cases of hyperplastic goitre. They were added 1 ml of lysis solution containing 10 *μ*l Tris-HCl pH 8, 1 M, 10 *μ*l EDTA 0.5 M, 25 *μ*l SDS 20%, 20 *μ*l proteinase K 10 mg ml^−2^, and were incubated at 37°C overnight. DNA was extracted using phenol–chloroform method.

### Bisulphite-PCR methylation analysis

Sodium-bisulphite modification of genomic DNA and PCR were performed according to Frommer's method (Frommer *et al*, 1992). Bisulphite causes deamination of cytosine that is transformed into uracyl (thymine) unless the cytosine is methylated, in this case it remains as cytosine. Briefly, 8 *μ*g of genomic DNA was digested with 10 U of *Eco*R1 (PROMEGA) for 1 h at 42°C. DNA was purified using phenol–chloroform–isoamylic alcohol, precipitated using ethanol and sodium acetate and resuspended in water. It was denatured with 3 M NaOH for 20 min at 42°C, treated with 3 M sodium bisulphite (SIGMA-ALDRICH, St Louis, MO, USA) (pH 5) and 10 mM hydroquinone for 18 h at 55°C. After treatment, DNA was purified using a Wizard DNA Clean-up kit (SIGMA-ALDRICH, St Louis, MO, USA) and desulphonated with 0.3 M NaOH and neutralised with 3 M ammonium acetate. To bisulphite-treated DNA, 10 *μ*g of glycogen was added, precipitated with ethanol and resuspended in 20 *μ*l sterile distilled water. PCR amplification was performed with 5 *μ*l of treated DNA. The sequence of interest in the bisulphite-treated DNA was amplified with bisulphite-specific primers: (sense) 5′ GGT TGT GTT AAT TTT AGA TT 3′, (antisense) 5′ ACT ACC CTA CCA ATA ACT CA 3′. The specific PCR product was 380 bp.

### Bisulphite sequencing

Amplified bisulphite-PCR products were subcloned into TA vector system (Invitrogen, San Diego, CA, USA), according to the manufacturer's instruction. Single colonies were amplified according to the manufacturer's instructions. DNA sequence analysis was carried out by automated DNA sequencers (Applied Biosystems, Foster City, CA, USA) using Big Dye Terminator Version 1 (Applied Biosystems). In all, 10 independent clones per case were analysed. Bisulphite treatment efficiency was proven by the complete conversion of the C to T in all sequences analysed.

## RESULTS

The pattern of expression of Met protein was investigated in frozen sections of 137 thyroid samples (Table 1

**Table 1:** Immunohistochemical expression of Met protein and methylation of MET promoter in papillary carcinoma and in other pathological conditions of the thyroid

**Histology**	**No. cases**	**Age mean**±**s.d.**	**Sex F/M**	**Met-positive cases**	**MET promoter methylation**
Papillary carcinoma	61	41±12	49/12	61	0/6^b^
Peritumoral normal thyroid tissue	45			−/+^c^	0/6
Insular carcinoma	3	42±20	2/1	3	nd
Anaplastic carcinoma	3	67±2	3/0	3	nd
Follicular carcinoma	4	34±6	3/1	4	nd
Oncocytic carcinoma	4	47±13	4/0	2/4	nd
Medullary carcinoma	1	51	0/1	0	nd
Follicular adenoma	20	45±14	14/6	4/20	nd
Thyroiditis	3	49±5	3/0	2/3	nd
Nodular hyperplasia	38	52±11	32/6	7/38	0/2

a Frozen sections were immunostained with DO24 mouse monoclonal antibody.

b Methylation status of 43 CpGs of the Met promoter in six cases of papillary carcinoma, in the corresponding normal thyroid tissue, and in two cases of hyperplastic goitre was investigated. Sodium bisulphite modification of genomic DNA and PCR were performed according to Frommer's method (see Materials and Methods).

c A weaker staining was present in the rim of peritumoral normal follicles with tall epithelium; whereas normal follicles with flat epithelium were not stained.

nd=not determined.

). A marked reactivity for the protein was observed in tumour cells of 61 out of 61 cases of papillary carcinoma (Figure 1A

**Figure 1:**
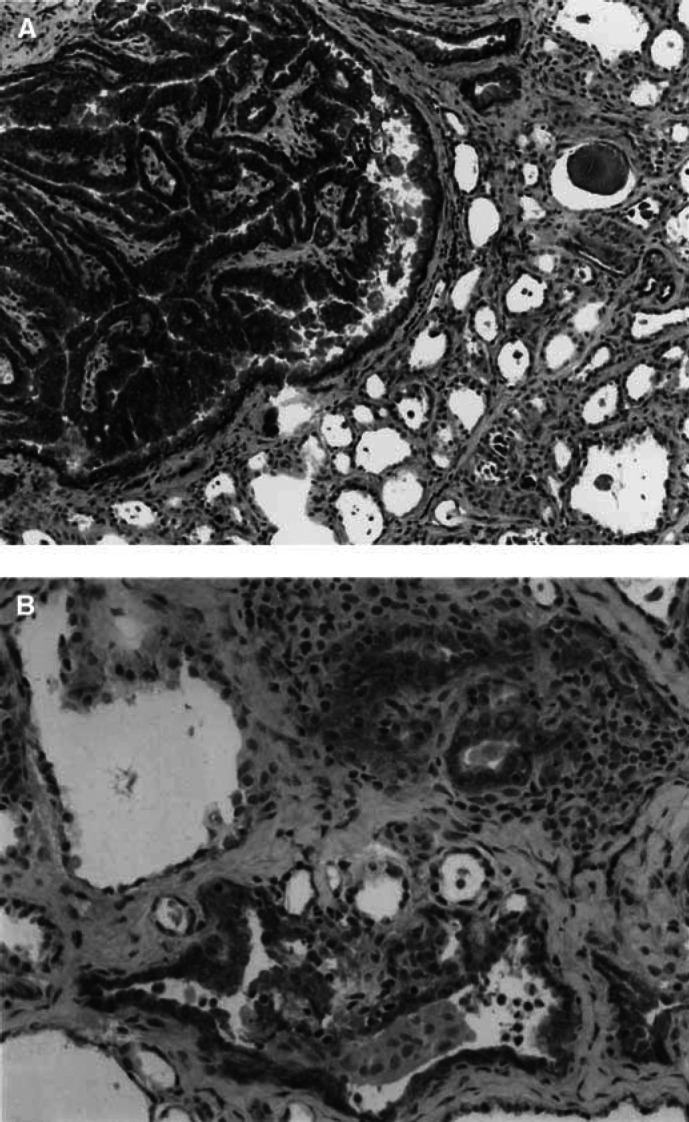
(**A**) Papillary carcinoma of the thyroid immunostained for Met protein with DO 24 monoclonal antibody. The tumour is intensely and diffusely positive; the peritumoral normal thyroid follicles are not stained (× 100). (**B**) Chronic thyroiditis immunostained for Met protein. Only those follicles infiltrated and surrounded by inflammatory cells are stained (× 250) (ABC-peroxidase, counterstained with haematoxylin).

); a much weaker staining was present in the rim of peritumoral normal follicles with tall epithelium, whereas normal follicles with flat epithelium were not stained. Met protein expression was investigated in frozen sections of 76 thyroid samples involved by pathological conditions other than papillary carcinoma. Among tumours, membrane staining for Met protein was observed in two out of three insular carcinomas, in two out of three undifferentiated carcinomas, and in one out of four Hürthle cell tumours. A weak cytoplasm staining was observed in four follicular carcinomas, and in four out of 16 follicular adenomas. In non-neoplastic conditions, a marked expression of Met protein was observed in follicles embedded in a chronic inflammatory reaction (Figure 1B), whereas weak staining was observed in tall cell follicles of seven out of 38 hyperplastic goitres.

**Figure 2:**
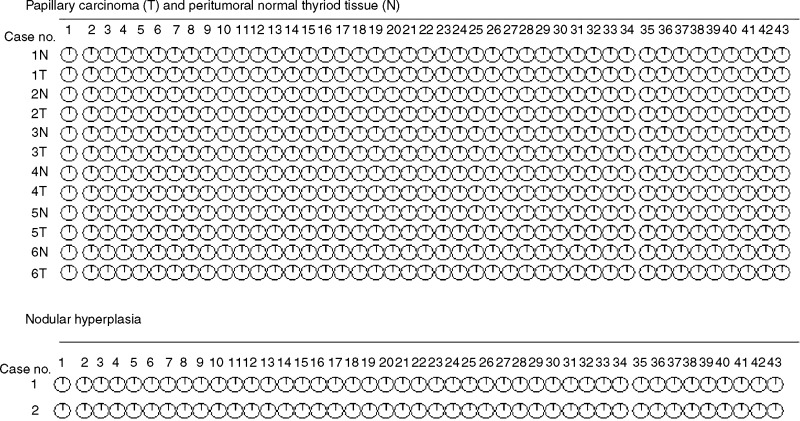
Methylation status of 43 CpGs of the Met promoter in six cases of papillary carcinoma, in the corresponding normal thyroid tissue, and in two cases of hyperplastic goitre was investigated. Sodium-Bisulphite modification of genomic DNA and PCR were performed according to Frommer's method (see Materials and Methods). In all, 10 colonies were analysed for each sample. Black and white areas represent methylated and unmethylated CpG sites, respectively.

The possibility that methylation is involved in the regulation of MET transcription was investigated through the analysis of the methylation status of 43 CpGs in six cases of papillary carcinoma, in the corresponding normal thyroid tissue, and in two cases of hyperplastic goitre (Figure 2

). Evidence of methylation was not found in any of the analysed CpG.

## DISCUSSION

In the present study, we provide further evidence that Met protein is highly expressed in papillary carcinoma cells, whereas it is absent or poorly expressed in normal thyroid follicles; moreover, we demonstrate for the first time that the different patterns of expression are not due to an altered methylation status of the MET promoter.

The rationale for our study derives from previous reports showing that hypomethylation is a kind of molecular mechanism leading to promoting high expression of oncogenes that encodes for some proteins with tyrosine kinase activity (Clark and Melki, 2002). They include several members of the Eph family of receptor tyrosine kinases (RTK) (Dottori *et al*, 1999), the c-fms oncogene that encodes for CSF 1R (Cui *et al*, 2001), and the erbB2/neu (Zhou *et al*, 2001).

Finally, in other studies on papillary carcinoma of the thyroid it was shown that abnormal methylation may occur in tumour cells, and is probably responsible for loss or for decreased expression of several genes including TSH receptor (TSHR) (Xing *et al*, 2003a) the Pendred syndrome gene SLC26A4 (Xing *et al*, 2003b), the Ras association domain family 1A gene (RASSF1A) (Schagdarsurengin *et al*, 2002), the metallothionein heavy metal binding protein gene (MT1G) (Huang *et al*, 2003) and the high-affinity cellular retinoic binding protein (CRABP1) (Huang *et al*, 2003).

Our findings strongly suggest that molecular mechanisms other than hypomethylation of the gene are responsible for the high expression of Met protein in papillary carcinoma of the thyroid. So far, it has been demonstrated that insertion of activated RAS and RET in normal thyroid cells causes upregulation of MET transcription (Ivan *et al*, 1997). The frequent occurrence of RET rearrangements in papillary carcinoma (Elisei *et al*, 2001; Soares *et al*, 2003) and the recent observation that a consistent number of nonrearranged cases have an activating mutation of BRAF that also cause signal transduction through the RET–RAS pathway (Fukushima *et al*, 2003) are consistent with the possibility that dysregulation of MET transcription is caused by the genetic transforming alterations specifically associated with this histotype. In addition, it was recently shown that tumour hypoxia may cause an increased transcription of MET through the upregulation of the hypoxia inducible factor-1 (HIF-1), which has two binding sites on the MET promoter (Pennacchietti *et al*, 2003). In a recent study, we have reported that HIF-1 is upregulated in tumour cells of most cases of papillary carcinoma and that histological alterations suggestive of a hypoxic condition are frequently present in this specific tumour (Scarpino *et al*, 2004).
